# Mental Health Awareness and Stigma in the General Population: A Mixed-Methods Approach in Semi-Urban Areas

**DOI:** 10.7759/cureus.100401

**Published:** 2025-12-30

**Authors:** Kaskar Alina Vasim, Purohit Kevin Ashokbhai, Birupaksha Biswas, Suklam Soham, Divyangkumar Patel, Shraddha Gurha

**Affiliations:** 1 Department of Medicine, Glocal University, Saharanpur, IND; 2 Department of Physiology, Gujarat Medical Education and Research Society (GMERS) Medical College, Vadnagar, IND; 3 Department of Pathology, West Bengal University of Health Sciences, Kolkata, IND; 4 Department of Medicine, University of Dhaka, Dhaka, BGD; 5 Department of Community Medicine, Dharmsinh Desai University, Nadiad, IND; 6 Department of Public Health, National Institute of Medical Sciences (NIMS) and Research, Jaipur, IND

**Keywords:** awareness, equity, mental health, semi-urban, stigma

## Abstract

Mental health conditions are one of the most common causes of disability in the world. However, stigma and a lack of awareness still influence the rate of recognition and seeking care, not to mention recovery. Although much has been done in relation to urban and rural populations, semi-urban communities, settlements characterised by intermediate population density, mixed livelihoods, and limited specialist services, are underrepresented. The gap in this review is filled by looking at the role of awareness and stigma in semi-urban settings. The objectives were the knowledge and attitude level of mental health, discussion of the nature of the stigma that limits care, and synthesis of the qualitative data into the perceptions and experiences of the community members. A narrative mixed-methods synthesis of the published studies during 2015-2025 was carried out, including quantitative data on prevalence and determinants as well as thematic analysis of qualitative results. Findings indicate a peculiar situation of fake availability: semi-urban dwellers face half-baked access to the discourse of modern health but have no structural means to pursue it, generating ambivalence whereby illness is both medicalised and interpreted in supernatural or moral terms. Stigma was also affected by gender norms, generational differences, socioeconomic differences, media portrayals, and poor healthcare systems. The review offers a fresh conceptualization of semi-urban stigma as a hybrid category, and also identifies avenues towards school-based literacy, community involvement, and primary care integration of culturally sensitive and equitable mental health interventions.

## Introduction and background

Mental health is now a global health priority, and depression, anxiety, and schizophrenia are some of the top causes of disability in the world today [1]. The World Health Organisation (WHO) estimates one in eight people are mentally ill, and mental illness has been among the largest contributors to years lived with disability and premature deaths [2]. Despite the development of new diagnostic and treatment approaches, there is currently a treatment gap, especially in low and middle-income countries (LMICs). Low awareness and persistent stigma are two key barriers to early detection, seeking treatment, and continued care involvement [3]. Stigmatisation, contrary to being a single construct, is multi-level. It is expressed at the individual level as internalised shame and self-stigmatisation, at the social level as prejudice, stereotyping, and exclusion; at the structural level as discriminative policies and underfunded mental health services [4]. Link and Phelan proposed that stigma is a dynamic process involving labelling, stereotyping, and deprivation, which is enhanced by power imbalances and cultural ideals [5]​​​​. It is a complex process in which individuals may fail to recognize their symptoms or delay seeking medical attention, resulting in secrecy and delayed treatment [6]. Consciousness is an important countermeasure. It involves consciousness of symptoms, causes, current treatment, and attitudes which promote acceptance and support of mentally ill people [7]. Increased sensitization can enhance recognition, reduce misconceptions, and foster more sympathetic responses [8]. However, these factors are inseparably interconnected: stereotypes are reinforced by ignorance, while stigma suppresses open discussion and hinders efforts to raise awareness within the community [9]. For example, in many communities, people may see persistent sadness as laziness or believe that hallucinations are caused by spiritual punishment. These misconceptions show how lack of awareness and stigma reinforce one another and cause a delay in help-seeking [10]. 

**Figure 1:**
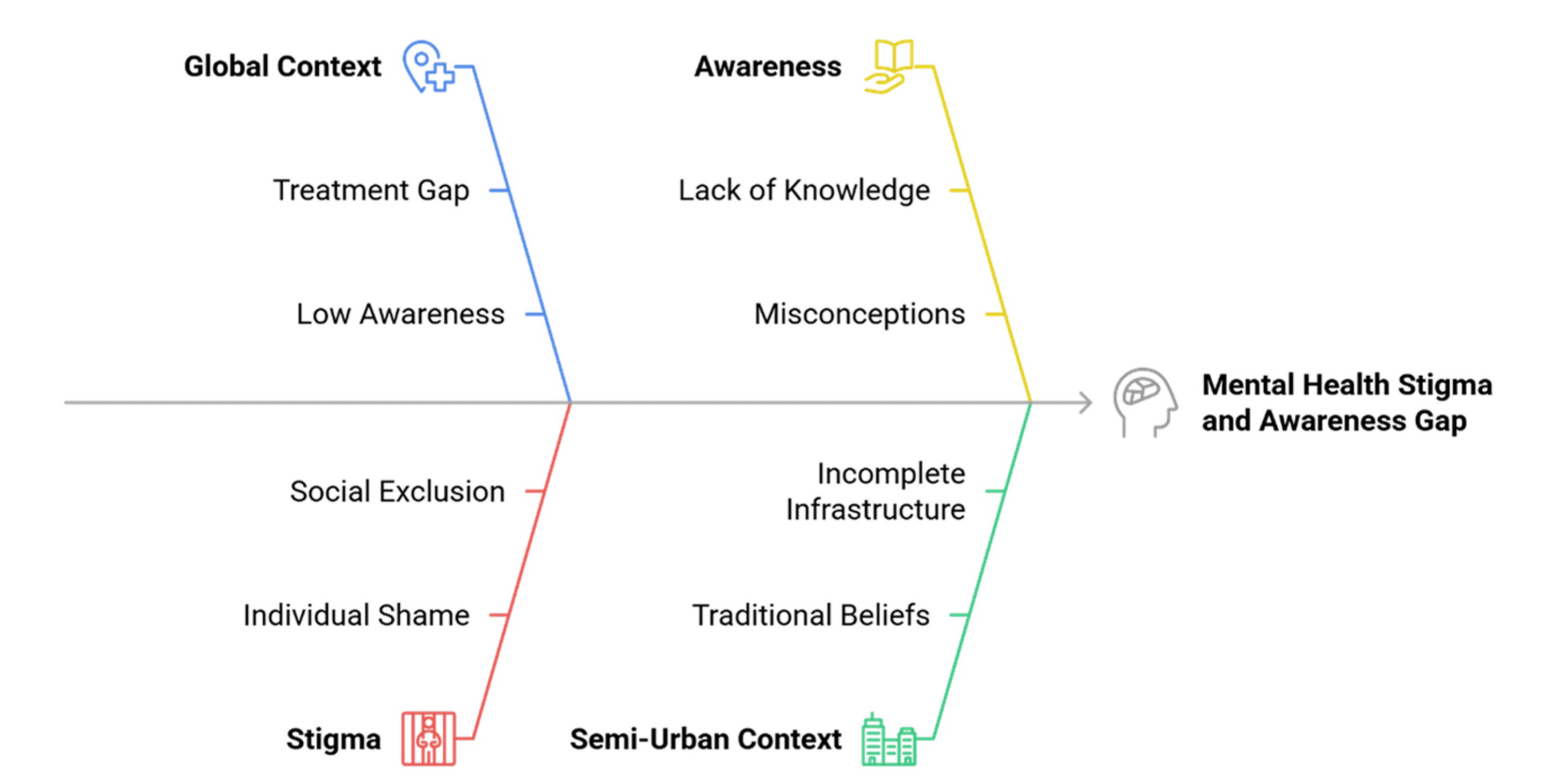
Determinants of Mental Health Awareness and Stigma in Semi-Urban Communities Created by authors

One particularly understudied area of this discussion is the semi-urban zone. These areas fall between rural and urban areas and are transitional areas with moderate population density, incomplete infrastructure, and socioeconomic heterogeneity [11]. Compared to urban centres, where services and campaigns are more accessible, or rural areas, where a lack of them is well-documented, semi-urban areas struggle with two problems. Although modern health information is increasingly disseminated through education and the media, many individuals continue to adhere to traditional belief systems that interpret mental illness as a spiritual weakness or a moral failing [7]. For instance, a young adult may learn online that depression is a treatable condition, yet family members may insist that it is a sign of personal failure or poor character [2]. Health centres are primitive in nature, but do not have special psychiatric care. This middle position creates special conditions whereby consciousness may be incomplete, stigma may be reinforced by both custom and falsehood, and health-seeking behaviour may be volatile [12]. These mixed signals create confusion, making individuals unsure whether to seek professional help or rely on traditional explanations. Figure 1 illustrates the determinants of awareness and stigma in semi-urban contexts.

Mental health awareness and stigma in urban or rural settings have been examined primarily in current studies [13]. It is claimed that these urban regions are overstretched and more likely to seek healthcare than the rural regions that are claimed to be under-resourced and that are in a state of constant cultural subjugation. Semi-urban populations often remain overlooked, and there is still a lack of understanding regarding the relationship between awareness and stigma in communities undergoing significant socioeconomic changes. Additionally, many studies rely solely on either quantitative surveys or qualitative case studies. While quantitative research provides valuable correlations, it tends to lack depth and generalizability, limiting our overall understanding of these issues. As a result, many existing findings show only one side of the issue. Quantitative studies may capture prevalence but cannot explain cultural meanings, while qualitative studies may describe experiences without indicating how common they are [14,15]. Using both forms of evidence together can provide a clearer and more complete understanding of how awareness and stigma operate in semi-urban settings, where people often balance traditional beliefs with new forms of health information [8].

This review is inspired by two important factors. First, it fills a critical research gap by comparing semi-urban populations that deal with unique problems due to the presence of mid-level social formations and partially developed medical facilities. These transitional settings are often overlooked in global mental health research, yet they reveal unique patterns of awareness and stigma that warrant systematic analysis. For example, residents may be aware of mental health concepts from schools or media, yet still rely on traditional explanations because services are limited or inaccessible. Second, the review adopts a mixed-methods approach, integrating quantitative evidence to capture measurable trends with qualitative insights that illuminate lived realities. This approach was chosen because semi-urban environments show complex behavioural patterns that cannot be understood through numbers alone; personal stories and community perceptions help explain why people delay or avoid care even when awareness exists. This dual lens strengthens the capacity to design timely and context-sensitive interventions. Beyond advancing scholarship, the review seeks to inform policymakers, practitioners, and community stakeholders by translating complex evidence into actionable strategies. In doing so, it underscores the importance of promoting equity in mental health care through enhanced awareness, reduction of stigma, and the creation of supportive environments that bridge the gap between research and practice.

Objectives of the review

The problem of mental health awareness and stigma among semi-urban residents is poorly investigated and will be considered in the current review. It attempts to determine the extent of knowledge, symptom awareness, and mental illness attitude, and also considers the type of stigma: self, social, and structural stigma that hinder care. Moreover, it converts qualitative knowledge about social perceptions and lived experiences into a community and incorporates the results into a mixed-methodology framework to produce actionable evidence. The ultimate objective will be to sensitize the transitional communities on culturally-relevant interventions and policies to reduce stigmatisation, increase awareness, and access mental health services.

Methodological considerations

A narrative mixed-methods review was conducted to examine mental health awareness and stigma in semi-urban populations, focusing on studies published between 2015 and 2025. The review synthesised a decade of global attention to mental health within the context of the Sustainable Development Goals and the WHO Mental Health Action Plan. A structured but non-systematic search was carried out in PubMed, Scopus, Web of Science, and PsycINFO using controlled terms and keywords such as mental health awareness, stigma, and semi-urban. The Boolean combinations used included “mental health awareness” AND “semi-urban,” “stigma” AND “transitional communities,” and “mental illness attitudes” OR “help-seeking” AND “semi-urban populations.” Studies were included if they examined awareness, stigma, or both within semi-urban or transitional settings. Additional inclusion criteria were publication in English; empirical design (quantitative, qualitative, or mixed methods); and clear reporting of semi-urban or peri-urban characteristics. Exclusion criteria included editorials, commentaries, studies not specifying semi-urban settings, and papers without extractable data on awareness or stigma.

Two reviewers independently screened titles and abstracts, followed by full-text screening to ensure eligibility. Disagreements were resolved through discussion, and a third reviewer was available for arbitration when needed. Quantitative findings were summarised narratively to present prevalence patterns, sociodemographic associations, and determinants of awareness and stigma, while qualitative findings were synthesised through thematic analysis to capture cultural narratives, community perceptions, and lived experiences not reflected in survey data. The qualitative synthesis followed an inductive thematic approach in which codes were developed line by line, grouped into categories, and refined into overarching themes. Coding consistency was ensured by cross-checking a subset of studies among reviewers. 

No formal statistical synthesis (such as meta-analysis, meta-regression, or logistic regression) was conducted because the included studies varied widely in design, outcome measures, and sample characteristics, and individual-level data were not available. Quantitative statistics such as p-values, confidence intervals, and effect sizes were extracted and reported only when provided in the original studies. Where such indicators were absent, the findings were summarised using ranges, percentages, and descriptive patterns. Missing or incomplete quantitative data could not be imputed or re-analysed and were therefore described narratively with acknowledgment of potential uncertainty. This approach was chosen to maintain accuracy while respecting the heterogeneous nature of the evidence base.

As this is a narrative review rather than a systematic review, PRISMA procedures were not strictly applied; however, methodological transparency was ensured by considering potential sources of bias, variations in definitions, and language limitations to maintain relevance and validity for semi-urban contexts. Bias was minimised by comparing patterns across multiple databases, including studies from diverse LMIC and transitional settings, and triangulating quantitative and qualitative evidence to avoid overreliance on any single study design. A brief coding framework and search term list are available upon request to enhance reproducibility. The limitations of narrative statistical reporting, including restricted precision and generalisability due to lack of pooled analyses, were recognised and incorporated into the interpretation of findings.

## Review

Global burden of mental health disorders

Mental disorders represent one of the largest health problems in the world, and the WHO reports that in 2019, 970 million people had a recognizable mental health concern [16]. A combination of depression and anxiety is estimated to contribute almost a third of disability-adjusted life years (DALYs) attributed to mental illness [17]. Suicide has been one of the major causes of premature deaths, as it is the fourth leading cause of death in people aged 15-29 years [3]. The numbers are meant to explain how big the problem is and how generational and social a crisis the untreated mental health issues are in the long term. Urgency is brought about by the economic aspect. The annual cost of lost productivity in the form of absenteeism, presenteeism, and dropout of the workforce alone is estimated to be over US$1 trillion per year [18]. In LMIC semi-urban settings, these economic losses are amplified because many families rely on single incomes and informal daily-wage work, making any mental-health-related impairment an immediate financial shock. These disruptive forces spill over to schooling, diet, and social stability in semi-urban families who, in most instances, rely on informal work or earn a single income.

Treatment gaps are not bridged by global policy advances. In the majority of the low and middle-income countries (LMICs), 75% of mentally ill individuals are not treated formally [19]. This gap is particularly evident in semi-urban regions, where primary health centres often lack adequately trained personnel, and families frequently delay seeking formal care due to financial constraints, geographic barriers, or the belief that symptoms will resolve spontaneously. While poor infrastructure, high costs, and shortages of human resources contribute to delayed care, psychosocial factors, especially lack of awareness and stigma, are critical determinants of symptom recognition and willingness to seek help [6]. The solution to the mental illness burden can therefore not be just the empowerment of the systems, but also the cultural or social stigmas that cause underutilization of services. An example of such dilemmas is semi-urban populations [20]. They are intermediate between rural underdevelopment of resources and urban over-concentration of services, and are characterized by moderate population density, rural-urban livelihoods, and underdeveloped but developing infrastructure. Locals might receive a health communication via online platforms or at school, but they do not have local facilities to implement the information. For instance, communities may be aware that depression can be treated, yet the nearest psychiatrist may be several hours away, reinforcing delays and reliance on non-medical explanations. Semi-urban environments, in their turn, underline the ways in which epidemiological burden intersects with fragmented systems and inaccessibility to sociocultural resources, which receive certain emphasis in the global health discourse [21].

Theoretical frameworks on stigma

Theoretical lenses are needed to break down the process, maintenance, and interaction of stigma with structural inequalities. According to Corrigan, there are three levels of stigma, including public and self-stigma, and label avoidance [21]. Public stigma means bias and prejudice against mentally ill people; self-stigma means the internalisation of the bias, resulting in low self-esteem; label avoidance means unwillingness to be treated because of the fear of the label of being mentally ill [9]. All these processes show that stigma discourages the use of services at different levels. In unequal power relations, Link and Phelan postulate stigma labelling, stereotyping, separation, and loss of status, which is reinforced [5]. This framework is concerned with institutional and structural dimensions of stigma, such as the absence of mental health service investment or health practice discrimination. Structural stigma can be found particularly in semi-urban settings where formal services are restricted and are of low priority [22,23].

**Figure 2:**
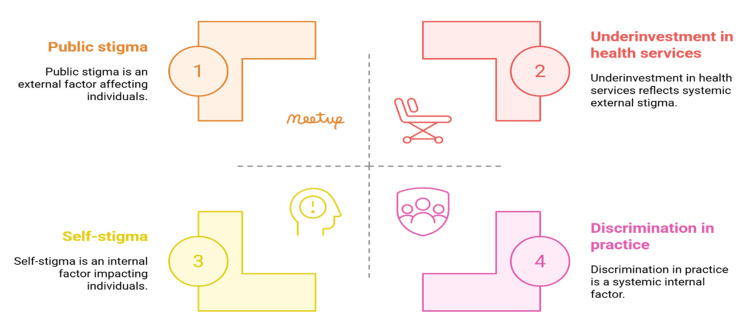
Framework of Stigma Processes and Structural Barriers in Mental Health Created by authors

These have been combined with other schools of thought, such as the labeling theory and the social identity theory [10]. The labelling theory is concerned with the effects of being stigmatised as mentally sick on social norms and opportunities, and the social identity theory elucidates the stigma as a function of the relations between the in-group and out-group. In semi-urban contexts where communities are closely knit, these theories manifest more sharply because individuals are easily identifiable, and gossip or reputational harm spreads quickly. Reputational damage is more dangerous in labelling that empowers concealment and silence [24]. The use of these frameworks underlines the reasons stigma is especially rooted in semi-urban areas. Traditional accounts of illness support public stigma, community surveillance supports self-stigma, and structural stigma is reflected in chronic underinvestment in health facilities [25]. These layered processes mean that stigma cannot be reduced through information alone but requires addressing community norms and improving the availability and quality of services. These findings justify the notion that information dissemination should not be the sole basis of anti-stigma interventions. They must also seek to change the minds of the masses, empower people, and bring a change in institutions to make people conscious of the sustainable change [26]. Figure 2 demonstrates how stigma manifests across personal and institutional domains, shaping access to and use of mental health care in semi-urban areas.

Mental health awareness, knowledge and attitudes

The sensitization is an extremely helpful outcome determinant, as it defines the knowledge regarding the symptoms, the cause beliefs, and the treatment willingness. It has been revealed in the 2015-2025 trends that the general population is currently sensitized about common ailments such as depression and anxiety, and it is largely due to global campaigns and online media [11]. But awareness of serious disorders such as schizophrenia and bipolar disorder is still insufficient, and psychotic symptoms tend to be explained by supernatural or moral factors in semi-urban populations [27]. In many LMIC semi-urban settings, hearing voices may still be attributed to spirit possession or a moral failing, which prevents families from seeking timely clinical support. The end product of these myths is stigmatisation as a barrier to early intervention [28]. Recognition of symptoms is intermittent. More likely is the fact that the explicit symptoms, such as sadness, fatigue, and excessive anxiety, are more well-known, but less conspicuous symptoms, such as cognitive diminution, disoriented thinking, or social withdrawal, are generally ignored [13]. These gaps often lead families to wait until symptoms escalate, after which referral is delayed due to reliance on informal providers like faith healers or traditional practitioners. This is compounded by late diagnosis and informal health care providers who may be a source of stigma or delay in clinical referral. These actors also demonstrate why partnerships with culturally trusted figures may be more effective than attempts to replace semi-urban mental health practices entirely.

Ambivalence is expressed in attitudes toward treatment. Professional care is open to younger residents who are more digitally connected. Gossip and discrimination may occur, but there is a disincentive to disclosure [29]. The fact that gender complicates this even more: women can empathize, yet they cannot act or get financial aid, and men are trained to be strong, and when they require help, it is perceived as an expression of weakness [15]. This combination often results in women hiding symptoms to maintain family harmony and men avoiding care to preserve social standing. Notably, awareness and stigma do not necessarily go in opposite directions. In certain semi-urban settings, the familiarity with diagnostic labels has strengthened negative stereotypes, for example, linking schizophrenia with violence [30]. This illustrates the paradox that increased exposure to mental health terms does not automatically reduce stigma and may reinforce harmful associations. This paradox is why awareness campaigns cannot be limited to fact-sharing. They should break the terrible stereotypes, create a human connection to lived experience, and present relatable narratives that reduce fear [31]. The best plans in semi-urban areas are the integration of mental health education in schools, community mobilisation of religious leaders as a means of legitimising biomedical care, and local media dissemination of information [32]. These interventions recognise that awareness is an active process that reshapes attitudes, challenges cultural narratives, and creates the conditions for early recognition and stigma reduction.

Stigma in semi-urban contexts

Stigma in semi-urban contexts is shaped by unique interactions between cultural expectations, social visibility, and limited healthcare infrastructure. Semi-urban areas lie between the city and the countryside and are peculiar in their dynamics [33]. In this review, semi-urban is defined as settlements with intermediate population density (20,000-100,000 residents), mixed rural-urban livelihoods, and intermediate infrastructure, where primary healthcare might be available but where there are few specialist psychiatric services [34]. The stigma is defined as a hybridity between the countryside, where stigma is rooted in traditional belief systems, and the metropolis, where stigma may persist despite widespread awareness campaigns. Older models have been merged with biomedical explanations of depression and anxiety [2]. This blend of traditional beliefs and partial biomedical understanding creates a form of ambivalent stigma in which individuals may recognise an illness but still attribute its cause to moral or supernatural factors. Such contradictions make the question of when and where to get care questionable.

Semi-urban social life contributes to stigma as well. The communities are small enough that reputational concerns become significant, and large enough that rumours can spread widely. Individuals are generally concerned that coming out will affect future employment or marital prospects [35]. For example, a young person experiencing anxiety may avoid seeking help because relatives fear it could affect marriage negotiations. This fosters concealment, particularly among families sensitive to social status. Meanwhile, since labour markets require largely informal working conditions in which physical proximity and perceived trustworthiness are critical factors, the so-called mentally ill can be pushed to the margins of economic life [36]. This exclusion reinforces both self-stigma and public stigma, creating a cycle in which people avoid care to protect their livelihood while worsening their mental health. This irony is the peculiar feature of semi-urban stigma: partial access to the discourses of modernity and no routine access to supportive mechanisms. There is mental health awareness, but the local systems are not acting on it, which leads to frustration, silence, and further stigmatisation [7]. This creates what reviewers called “false availability,” where information is present but services are absent, producing confusion and mistrust. The interventions need to make this visible in ways that disorient cultural explanations and highlight system gaps. Table 1 illustrates the comparative dimensions of mental health stigma, highlighting the peculiar ambivalence in semi-urban settings.

**Table 1:** Urban, Rural, and Semi-Urban Contrasts in Mental Health Stigma.

Dimension	Urban Context	Rural Context	Semi-Urban Context	Reference
Population & Structure	Dense, metropolitan, with access to campaigns	Sparse, traditional, limited access	Intermediate density (20,000–100,000), mixed livelihoods, partial infrastructure	[2]
Beliefs about Mental Illness	Biomedical explanations are dominant, and awareness campaigns present	Traditional/spiritual interpretations dominate	Ambivalence: biomedical recognition + supernatural explanations	[20]
Healthcare Access	Specialist psychiatric services are available	Very limited, primary care, or none	Primary care present, but few specialists → faked availability	[15]
Social Life & Stigma	More anonymity, less reputational risk	Strong social control, traditional community sanctions	Small enough for reputational harm, big enough for rumours to spread widely	[3]
Economic Impact	Larger formal job markets	Agriculture, subsistence-based	Informal labour markets → mentally ill marginalized	[11]
Cultural/Social Norms	Awareness campaigns shape perceptions	Deeply rooted traditional belief systems	Contradictions: exposure to modern discourses, but a lack of supportive systems	[19]
Key Effect	Stigma shaped by awareness & competition	Stigma rooted in cultural beliefs	Ambivalent stigma: awareness exists, but contradictions + systemic gaps fuel secrecy and silence	[6]

Gender and generational differences in awareness and stigma

Gender roles shape both awareness and the lived experience of stigma. Women in semi-urban areas, often occupying caregiving roles, demonstrate greater recognition of symptoms in others [37]. Yet when they themselves experience mental illness, stigma is harsher. We can measure the symptoms by failure to meet household chores or signs of weakness [5]. Even the women themselves cannot access formal care as they are not only financially dependent but cannot even move without being unaware of the treatment possibilities. In many LMIC semi-urban contexts, women may avoid seeking help because travelling alone to clinics is discouraged or because disclosure may threaten family harmony. Men are pressured in other ways. The nature of resilience and productivity can be explained more in semi-urban economies that are based on manual labour or small businesses [38]. Distress can be viewed as something that is not permitted to exist alongside these roles, and men are not encouraged to recognise the symptoms or seek assistance [6]. As a result, men often suppress emotional distress to maintain social expectations, which delays recognition and intensifies self-stigma. Nevertheless, research demonstrates that younger men who are exposed to digital campaigns are more likely to talk about mental health and that norms shift [27]. Such an awareness of variability delegitimizes the desire to think about the attitudes of males so casually and makes the intervention a possibility.

There is a generational divide, which is an added dimension. The younger age groups that are more exposed to schools and social media are more aware of disorders such as depression and anxiety [39]. Being aware does not necessarily mean being accepting: not all youths stop using stigmatising terms or socially isolate themselves in relation to their peers with the condition [40]. This shows that knowledge alone does not eliminate discriminatory behaviour, especially when peer influence and cultural norms remain strong. The elderly can make sense of illness in a moral or even spiritual way, and this strengthens family silence [9]. Tensions like these in the context of multi-generational households, typical of semi-urban localities, may postpone care. For example, a young person recognising symptoms may still be overruled by elders who believe the condition reflects moral weakness or divine displeasure. What it means in terms of interventions is simple: the programmes should not only be gender-responsive but also intergenerational [14]. Women require empowerment programmes that can enable them to be mobile and financially independent. In the case of men, re-framing strategies that position help-seeking behaviour as a strength instead of a weakness may be appealing. Transference between generations and the establishment of positive environments within the family can be provided by the intergenerational psychoeducation of the school population that involves parents or community forums that engage the elders [41]. Such integrated approaches ensure that awareness gains among youth can translate into more supportive family-level norms.

Role of education and socioeconomic status

Educational attainment is one of the strongest predictors of mental health awareness. The more culturally developed of the semi-urban districts will be able to assist the population in recognising the symptoms more effectively and even facilitate the convenience of treatment by professional experts [42]. They also access the information available on the internet and become familiar with health campaigns [10]. Low literacy has also contributed to the maintenance of the myths regarding mental illness as contagious, incurable, and that to this day has remained incurable and continues to be incurable [11]. In many LMIC settings, limited schooling reinforces reliance on cultural myths, making families misinterpret treatable symptoms as character flaws or lifelong curses. The differences lead to the unequal interpretation of the same society, whereby one of the families embraces the medical paradigm, and the other family embraces the myths of the culture [43].

Education and socioeconomic status (SES) also overlap with stigma and access. Wealthier families may readily avoid domestic constraints, either by migrating to metropolises or by obtaining non-state assistance, which might be less stigmatised than state assistance [12]. But stigma exists too: it might be less obvious among more successful groups, like social distancing or muttering disapproval. Middle-income families are a very vulnerable group. They may have primitive knowledge and want to be helped, but cannot do it due to economic circumstances and substandard local services, and it frustrates and makes them bear pain secretly [13]. These families often remain trapped in a cycle of awareness without access, resulting in hidden distress and delayed treatment. Socioeconomically disadvantaged households are disproportionately affected, as financial constraints limit their ability to afford clinic visits and medications. In such settings, stigma may normalize neglect and perpetuate avoidance of care, reinforcing a vicious cycle of poverty and illness within the community [44]. This entrenches a cycle where untreated illness reduces income, and reduced income further limits access to care.

What arises is a gradient and not a binary. Stigma crosscuts across all socioeconomic categories but is expressed in different ways: as an affordability constraint among the poor, as a limited choice among the middle, and as a reputation among the rich [45]. This gradient indicates that interventions must be sensitive to each group’s barriers rather than assuming a uniform solution. The effects of policymaking are: mental health must form part of primary care to reduce the cost burden and family income, subsidize low-income family care, and develop campaigns that make sense to low-literacy groups [33]. Similarly, interventions should confront stereotypes within all groups of SES, so that the awareness is accompanied by social acceptance and structural support.

Influence of media and technology

Media and technology are among the most influential forces shaping mental health awareness and stigma in semi-urban areas. Digital platforms, community radio, and television create channels for education, but they also amplify stereotypes [46]. On the positive side, social media campaigns and online resources have increased exposure to mental health concepts, particularly among younger semi-urban residents with internet access [1]. These platforms allow youth to connect with global narratives that normalize help-seeking, offering counterpoints to traditional silence around mental illness. However, semi-urban populations often face digital divides. Limited connectivity, low digital literacy, and uneven access to smartphones restrict the reach of online campaigns [47]. Moreover, exposure without critical appraisal can reinforce stigma: psychiatric conditions are sometimes sensationalized in news stories, while television dramas present individuals with severe illness as violent or comic figures [31]. Such portrayals foster fear and ridicule, counteracting the benefits of awareness initiatives. These distortions are especially influential in LMIC semi-urban regions where television remains a primary source of information.

Nevertheless, context-sensitive approaches show promise. Community radio broadcasts in local dialects, participatory short films screened at town gatherings, and podcasts featuring lived-experience narratives have effectively reshaped attitudes in semi-urban regions [48]. These methods capitalize on the oral traditions and collective culture prevalent in such communities. Locally produced content often resonates more deeply than national campaigns because it reflects real community experiences and voices. The lesson is that technology and media are not inherently positive or negative; their impact depends on design, cultural tailoring, and the capacity of semi-urban residents to interpret and apply the information [20]. Effective strategies must therefore combine digital literacy programs with responsible journalism and locally rooted storytelling that reflects recovery, resilience, and inclusion. Table 2 highlights the contrasting impacts of media and technology in semi-urban settings, showing both their potential for awareness and their limitations due to digital divides and negative portrayals.

**Table 2:** Media and Technology in Semi-Urban Mental Health Awareness and Stigma.

Aspect	Positive Impact	Barriers	Context-Sensitive Strategies	Reference
Digital platforms (social media, online resources)	Increases exposure to mental health concepts; connects youth with global narratives	Digital divide (limited access, low literacy); misinformation; stigma amplified	Combine with digital literacy programs	[7]
Mainstream media (TV, news, dramas)	Potential for awareness campaigns	Sensationalism; portrayal of the mentally ill as violent/comic → fosters fear and ridicule	Encourage responsible journalism	[11]
Community-based media (radio, podcasts, local films)	Accessible, culturally tailored, participatory, and effective at reshaping attitudes	Limited funding; smaller reach compared to global platforms	Use local dialects, lived-experience storytelling, and town gatherings	[23]

Community perceptions and lived experiences

The prevalence rates could be presented in the form of quantitative data, and where the stigma experience and awareness are revealed in real life is the place. Mental illness is viewed in the context of social responsibility and morality in semi-urban communities [49]. Depression is considered as laziness and psychosis as spiritual infestation or ancestral disfavour [5]. Families often hide diseases because they are afraid of gossip that may ruin their lives or marriages. This sort of concealment maintains silence as well as delays in treatment. In many LMIC semi-urban settings, individuals may avoid visiting clinics because even being seen near a mental health facility can trigger rumours that affect marriage prospects or employment. The same is reflected in schools and places of work. Instead of seeking assistance, children with behavioural issues can be punished, and adults can be subjected to subtle marginalisation in workplaces that rely on informal work and human trust [50]. These patterns show that stigma is not merely an attitude but a social process that shapes access to education, economic opportunities, and participation in public life. These experiences suggest that stigma can be changed into actual drawbacks that restrict access to education and money [25].

Resilience and adaptation also come as a result of living experiences. Those and other South Asian women groups have established peer circles where they share their experiences of anxiety and depression to rebrand them as group issues and not individual failures [7]. Psychoeducation has been integrated with prayer in faith-based organisations in certain parts of Africa as it reduces stigma by legitimizing biomedical care and spiritual practices [18]. These hybrid models often succeed in semi-urban areas of low- and middle-income countries (LMICs) because they build on trusted community traditions while gradually introducing biomedical explanations. Such examples demonstrate how semi-urban societies can reinterpret prevailing narratives to support change when the advocates of that change are trusted local actors. Such lived experiences need to be captured so that effective interventions can be designed. They remind policymakers and practitioners that stigma is not merely externalized, but is internalized, negotiated, and even resisted within communities [29]. Programmes that mobilise and utilise the already existing strengths of the community, as opposed to substituting them, are more likely to be successful in terms of changing perceptions. This community-centred approach helps ensure that interventions do not feel imposed from outside but emerge naturally from local values and lived realities.

Healthcare system problems

Structural barriers are one of the most evident problems of semi-urban mental health. The concentration of specialised services is urban, and the underserved regions include rural areas, where, in fact, semi-urban zones lie between these two, and they are not considered in policy or planning [16]. The outcome is uneven service delivery. Primary care centres might be available, but they are usually ill-equipped, and the staff are not trained in mental health, and psychotropic medication is not provided regularly [18]. For example, a patient with severe depression in an LMIC semi-urban region may attend a clinic only to find that the provider cannot prescribe appropriate medication due to stockouts or lack of training. Task-shifting programmes, in which nurses or general practitioners are trained to treat common conditions, are promising, but lack sustainability. Their performance is limited by employee turnover, poor supervision, and workloads [7]. Moreover, physical infrastructure is often inadequate: semi-urban clinics may lack individual consultation rooms, which may lead to the unwillingness of people to disclose their personal mental health problems. The institutional stigma enhances these barriers [46]. Cases in semi-urban clinics in South Asia and Latin America, about patients being sent home or treated disrespectfully, support the suspicion of the community towards formal services [29]. Such negative experiences reinforce avoidance of care and strengthen beliefs that mental health systems cannot be trusted.

These experiences create a symbolic meaning that mental health is not valued and lead to stigma at the system and social levels. There are also policy gaps that are critical. National mental health plans are more likely to be included in primary care without budgeting or staffing sufficient resources to rural semi-urban regions [12]. The amount of money distributed to the tertiary hospitals within the urban areas and the remaining towns that are situated at the periphery is also not evenly distributed. This uneven distribution contributes to what researchers describe as structural invisibility, where semi-urban communities receive too little investment to build functional services yet too much population density to be treated like rural zones. Such oversight helps create what could be described as structural invisibility: semi-urban areas are not rural enough to be targeted by donors or urban enough to enjoy the benefits of concentrated investment [36]. Systemic change has to address such obstacles. Universal health coverage should be able to integrate mental health, but should clearly involve semi-urban clinics. Professional, social, financial, and incentives are required to lure trained personnel to these areas [4]. Without such measures, awareness campaigns may raise demand without delivering services, deepening frustration and reinforcing distrust [21]. A combination of strengthened primary care integration, consistent medication supply, and community-trusted providers is essential to close the treatment gap in these transitional regions. Table 3 summarizes the major structural challenges limiting effective mental health care in semi-urban settings, including service gaps, task-shifting difficulties, and institutional stigma.

**Table 3:** Structural Barriers to Mental Health Care in Semi-Urban Settings.

Barrier	Description	Consequence	Reference
Service Provision Gaps	Primary care centers exist, but are poorly equipped; psychotropic medication is irregular.	Inconsistent treatment availability	[9]
Task-Shifting Challenges	Nurses/GPs trained to manage conditions; limited by turnover, supervision, and workload	Unsustainable care, reduced trust	[15]
Physical Infrastructure	Lack of private consultation rooms in clinics	Discourages disclosure of mental health concerns	[30]
Institutional Stigma	Patients were dismissed or treated disrespectfully in semi-urban clinics	Reinforces community distrust of services	[11]
Policy & Funding Gaps	National strategies overlook semi-urban areas; funding is directed to cities.	Structural invisibility → clinics under-resourced	[3]

Interventions to reduce stigma and enhance awareness

Interventions in semi-urban areas must operate across multiple levels: individual, community, and structural. Evidence suggests that single strategies rarely suffice; multi-component approaches tailored to transitional contexts are most effective [24]. Public campaigns can be powerful when rooted in local culture. Community theatre, participatory storytelling, and visual media in local languages have reduced negative stereotypes in several semi-urban regions [14]. Such culturally grounded methods are especially effective in LMIC settings where trust in local messengers is stronger than in formal institutions. Yet these efforts are often project-based and lack sustainability once external funding ends. Embedding campaigns within existing community institutions such as schools, religious centres, and women’s associations enhances longevity. School-based programs are particularly promising. Integrating mental health literacy into curricula improves recognition and reduces stigma among adolescents [17]. When schools engage parents through workshops, the impact extends across generations, bridging awareness gaps. However, limited school attendance, teacher shortages, and resource constraints in poorer semi-urban districts may restrict the reach of these programs.

Peer support and advocacy by individuals with lived experience offer another effective pathway. These initiatives humanize mental illness, replacing stereotypes with personal stories that foster empathy [31]. NGOs often play a facilitative role, but reliance on external actors raises concerns about sustainability. Long-term change requires shifting leadership to local community groups and integrating peer support into routine public health services. Healthcare integration remains critical [43]. Training primary care providers in semi-urban clinics to recognize and treat common disorders ensures that increased awareness is matched with accessible services [37]. This is essential in LMIC semi-urban regions where specialised providers are scarce and primary care often serves as the first and only point of contact. However, challenges such as staff overload, lack of supervision, and limited medication supplies must be addressed to avoid disillusionment.

Interventions must also be critically appraised for limitations. Awareness campaigns can sometimes backfire by increasing recognition without reducing stigma, or by overwhelming fragile local health systems [31]. Peer-support groups may struggle to sustain momentum without resources. School programs risk excluding out-of-school youth, a significant population in semi-urban areas. Digital interventions may also unintentionally widen inequalities if they presume access to smartphones or stable connectivity. Acknowledging these limitations helps refine strategies to ensure interventions are realistic, culturally sensitive, and resilient over time [10]. Interventions in semi-urban areas should be layered, combining community engagement, educational reform, peer advocacy, and systemic investment [28]. Sustainable impact requires simultaneous efforts to address stigma, expand service capacity, build local leadership, and ensure financial and infrastructural stability. Equity in mental health care cannot be achieved through awareness alone; it requires simultaneous efforts to dismantle stigma and strengthen service provision.

Limitations and future recommendations

The scope of this review is constrained by notable limitations. Empirical data specific to semi-urban populations are limited, with many studies aggregating rural and peri-urban contexts, thereby obscuring unique patterns. Stigma is itself a limiting property of the quality of evidence, in that the prevailing fear of reporting is the usual source of selective reporting and underestimation of prevalence. Also, the bulk of the existing literature is cross-sectional and provides snapshots, not longitudinal views of changing awareness and stigma. Finally, the research publication language barrier may have contributed to the omission of locally relevant studies, thus restricting the cultural lens through which semi-urban processes were viewed.

It has also been suggested that the follow-up research, which would be carried out, is a longitudinal research and a mixed-method research where tendencies of prevalence would not only be observed, but also experienced in the transitional setting. The hybrid cultural forms of semi-urbanities and the infrastructures of the middle level require that the interventions be developed based on this duality. Telepsychiatry and mobile mental health apps are examples of digital tools that can be effective but will require some attention when digital literacy conditions are unequal. Similarly, participatory research led by communities needs to be scaled to take advantage of local agency and align with culture. Sealing these gaps will contribute to the body of policy and intervention evidence to improve equitable and stigma-free mental health care in semi-urban groups.

## Conclusions

The review illuminates the particularities of mental health awareness and mental health stigma within the semi-urban context, where transitional spaces are a complex of problems that cannot be replicated either in rural or urban settings. Unlike rural populations, where stigma is rooted in traditional beliefs, and urban ones, where awareness campaigns often exist without addressing structural discrimination, semi-urban populations are confronted with a paradox of false availability. The inhabitants are partly subjected to the new health discourses but not provided with the same services to follow them, becoming ambivalent. Mental illness is biomedicalized, but supernatural or moral factors explain serious manifestations. This review provides an in-depth perspective on the issue that tends to be neglected in one-method studies and integrates quantitative information with qualitative insights. We do not know of any of the earlier reviews that have attempted to conceptualise the semi-urban stigma systematically as an exposure without support hybrid construct. It prolongs the rural-urban dichotomy and transforms the conception of pseudo-availability, and provides a new theoretical orientation to the forthcoming studies. Until then, it has some useful things to say: awareness can be turned into action, which can be done by empowering primary care, by bringing awareness to schools and communities, and by basing campaigns on the grounds of cultural norms. Thus, the review fills an overlooked gap and identifies how equitable and stigma-free mental health care can be achieved among transitional populations.
